# Thrombectomy outcomes for acute ischemic stroke in lower-middle income countries: A systematic review and analysis

**DOI:** 10.1016/j.wnsx.2024.100317

**Published:** 2024-03-05

**Authors:** Jaims Lim, Alexander O. Aguirre, Abbas Rattani, Ammad A. Baig, Andre Monteiro, Cathleen C. Kuo, Manhal Siddiqi, Justin Im, Steven B. Housley, Matthew J. McPheeters, Shiau-Sing K. Ciecierska, Vinay Jaikumar, Kunal Vakharia, Jason M. Davies, Kenneth V. Snyder, Elad I. Levy, Adnan H. Siddiqui

**Affiliations:** a Department of Neurosurgery, Jacobs School of Medicine and Biomedical Sciences, University at Buffalo, Buffalo, NY, USA; b Department of Neurosurgery, Gates Vascular Institute at Kaleida Health, Buffalo, NY, USA; c Jacobs School of Medicine and Biomedical Sciences, University at Buffalo, Buffalo, NY, USA; d Department of Radiation Oncology, Tufts University Medical Center, Boston, MA, USA; e Department of Neurosurgery and Brain Repair, University of South Florida, Tampa, FL, USA; f Department of Bioinformatics, Jacobs School of Medicine and Biomedical Sciences, University at Buffalo, Buffalo, NY, USA; g Canon Stroke and Vascular Research Center, University at Buffalo, Buffalo, NY, USA; h Jacobs Institute, Buffalo, NY, USA; i Department of Radiology, Jacobs School of Medicine and Biomedical Sciences, University at Buffalo, Buffalo, NY, USA

**Keywords:** Disparity, Global, Ischemic stroke, Lower-middle income, Stroke, Thrombectomy, Worldwide

## Abbreviations and acronyms

AIS: acute ischemic stroke
CI: confidence interval
HERMES: Highly Effective Reperfusion Evaluated in Multiple Endovascular Stroke
ICH: intracranial hemorrhage
IV: intravenous
LIC: low income country(ies)
LMIC: lower-middle income country(ies)
mRS: modified Rankin Scale
MT: mechanical thrombectomy
NIHSS: National Institutes of Health Stroke Scale
PRISMA: Preferred Reporting Items for Systematic reviews and Meta Analyses
SWIFT: SOLITAIRE™ FR With the Intention For Thrombectomy
sICH: symptomatic intracranial hemorrhage
TICI: thrombolysis in cerebral infarction
tPA: tissue plasminogen activator
TREVO 2: Trevo versus Merci retrievers for thrombectomy revascularization of large vessel occlusions in acute ischaemic stroke
WHO: World Health Organization

## 1 Introduction

Stroke is one of the most debilitating injuries to the body and brain, accounting for significant morbidity and mortality as well as a financial burden on a nation's healthcare infrastructure.^1^ With advancement in technology and endovascular techniques, patients undergoing endovascular therapy for acute ischemic stroke (AIS) due to large vessel occlusions have experienced improved outcomes.^2^ However, all of the trials proving the efficacy of mechanical thrombectomy (MT) for stroke were conducted in high income countries.^1^^,^^3^, ^4^, ^5^, ^6^, ^7^ This presents a challenge in terms of a paucity of randomized data regarding the efficacy and utility of MT in low-income and lower-middle income countries (LIC and LMIC, respectively). It is estimated that 87% of all deaths worldwide and 81% of disability related to AIS occur in low- and middle-income countries.^8^ Several studies highlight significant disparities in access and care and worse stroke outcomes in LIC and LMIC compared to higher income counterparts.^9^, ^10^, ^11^ Poorer outcomes can be attributed to a variety of factors including lack of healthcare access, lack of imaging and interventional resources, prevalence of stroke comorbidities, and care infrastructures.^12^ Importantly, these poorer outcomes can be linked to lack of access to endovascular treatment capabilities and surgeons.^13^ To our knowledge, a review of the literature of thrombectomy for stroke and respective outcomes in LIC and LMIC has not been conducted. With calls to determine and elucidate the global burden of neurosurgical disease,^14^^,^^15^ we performed a review of thrombectomy outcomes in these countries to potentially further highlight the significant disparity in endovascular access and care in lower income countries.

## 2 Material and methods

Patient consent and institutional review board approval were not applicable for this systematic review and analysis. Data that support the findings are available from the corresponding author on reasonable request.

### 2.1 Article selection process

We performed comprehensive searches of PubMed and Embase databases from their earliest records to August 2022, following the Preferred Reporting Items for Systematic reviews and Meta Analyses (PRISMA) guidelines. The searches were performed using keywords combined with Boolean operators to capture relevant publications from World Bank Country Income Classification-designated LIC and LMIC (https://datahelpdesk.worldbank.org/knowledgebase/articles/906519-world-bank-country-and-lending-groups). Pertinent publications on AIS and MT were included. Exact keywords and search terms are provided in Table 1. All results were entered into Rayyan (http://rayyan.qcri.org), a mobile web application for systematic reviews and meta-analyses. Initial screening consisted of assessment of all abstracts for selection of studies in English that reported patients ≥18 years of age with AIS treated with MT. Case reports and series (≥5 cases) were included at this stage to make sure all reports in the countries of interest were captured. Animal, cadaveric, in vitro studies, technical reports, and background articles were excluded at this stage. The remaining abstracts were assessed. The initial screening of abstracts was independently performed by 5 authors. The full text article screening, if not a conference or meeting abstract, was then independently performed by these authors. Discrepancies were resolved by a third author. Our inclusion criteria were 1) patients with confirmed AIS treated with MT; 2) reports of procedural characteristics and recanalization rates; 3) details regarding periprocedural complications, including symptomatic intracranial hemorrhage (sICH); 3) reports of 90-day clinical outcomes following MT (modified Rankin scale [mRS] score and mortality). Studies meeting these criteria were assessed for institution and time period of patient enrollment, and those with overlapping patient populations were excluded while retaining the most complete or recent publication.

**Table 1:**
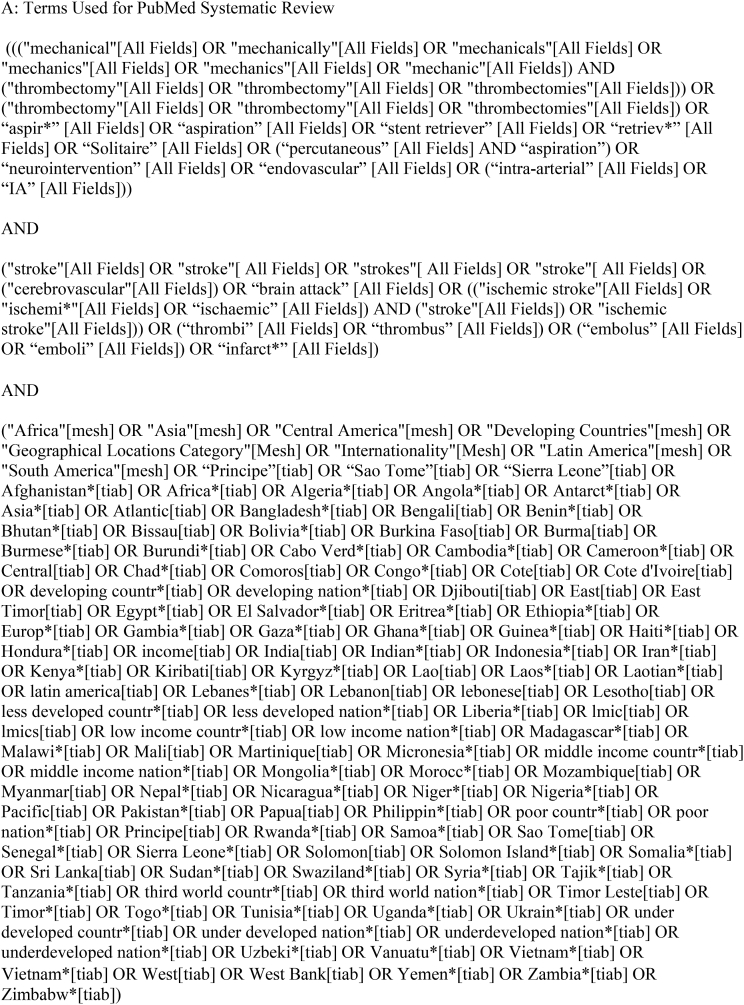

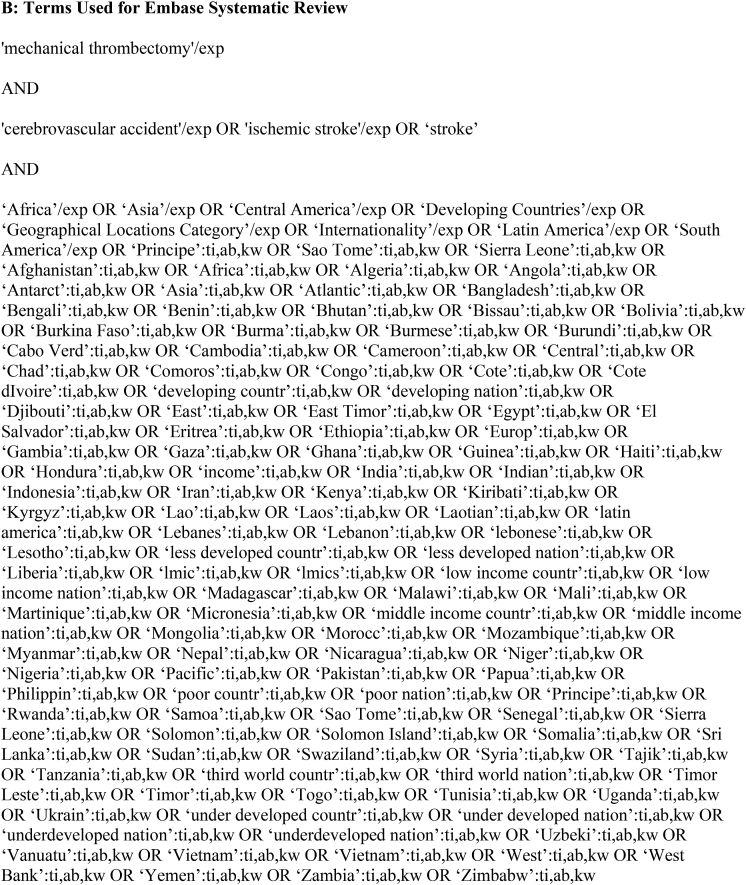
Search Strategies.

### 2.2 Data extraction

Variables of interest extracted from the included publications were total number of patients treated, mean or median patient age, proportion of patients receiving intravenous (IV) tissue plasminogen activator (tPA), vessel occlusion location, comorbidities, mean or median National Institutes of Health Stroke Scale (NIHSS) score at presentation, and mRS score at baseline. Procedural characteristics were also collected including symptom onset-to-door time, symptom onset to recanalization time, median or mean thrombolysis in cerebral infarction (TICI) recanalization rate. Reports and rates of complications, such as sICH and asymptomatic ICH, were collected. Outcomes data, including discharge mRS scores and in-hospital and post-hospital mortality rates, were also collected.

For quality assessment, we evaluated if individual studies had: a prospective design, stroke severity, procedural details (thrombectomy ± thrombolysis), descriptions of peri- and post-procedural complications, angiographic outcomes, clinical outcomes, and long term follow up (≥90 days). Each of the above is rated with a ‘yes’ or ‘no’. Studies having ≤2 ‘yes’ were considered low quality, with 3–4 ‘yes’ were co nsidered moderate quality and ≥5 were considered high quality studies (Supplementary Table 1).

### 2.3 Statistical analysis

Pooled estimates weighted for sample size were calculated for the variables of interest using the random effects model (DerSimonian and Laird approach), which accounted for within-study and between-study variances. To assess the heterogeneity among studies, the I^2^ statistic was calculated to reflect the percentage of variation among studies rather than chance alone. An I^2^ >50% was indicative of significant heterogeneity. We then generated forest plots to show the prevalence and estimated overall rates with 95% confidence intervals (CIs). Funnel plots to assess publication bias cannot be created as these are single arm pooled analyses. All statistical analyses were conducted using the RStudio “meta” function package (https://www.r-project.org/).

## 3 Results

### 3.1 Systematic search

**Fig. 1:**
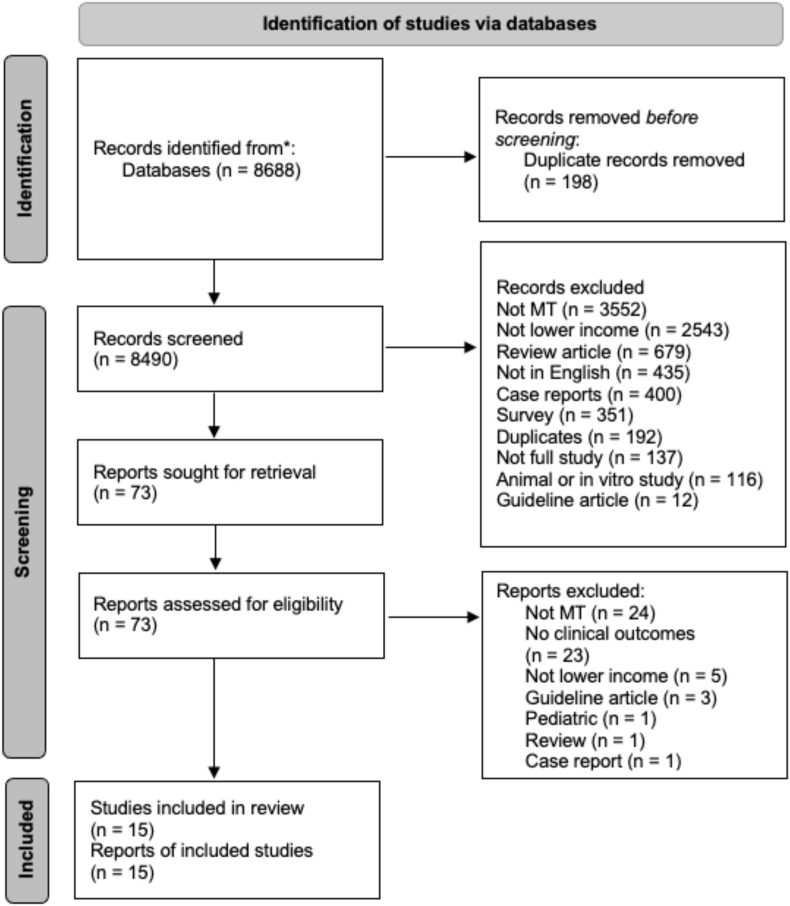
Preferred Reporting Items for Systematic reviews and Meta Analyses (PRISMA) review for mechanical thrombectomy (MT) publications from low income countries and low-middle income countries. Flow diagram illustrates the screening and identification of study articles and abstracts. *PubMed and Embase databases.

A total of 8688 studies were identified in our initial search. Of these, 198 were duplicates, resulting in 8490 unique articles and abstracts. After initial abstract screening, 8417 were excluded, and 73 were selected for full-text assessment. From these, an additional 58 were excluded. Seven full text articles^16^, ^17^, ^18^, ^19^, ^20^, ^21^, ^22^ and 8 abstracts^23^, ^24^, ^25^, ^26^, ^27^, ^28^, ^29^, ^30^ were included after final article selection. One of the articles (Mesiano et al) described two thrombectomy populations including one group that received both thrombolysis and thrombectomy versus thrombectomy alone with specific and separate patient data for each group.^19^ Thus, for data presentation and analysis, the patients were treated as two separate groups and studies. The search strategies and reasons for exclusion at each step are seen in Fig. 1.

### 3.2 Background of selected publications

The 15 included studies comprised a total of 1112 patients and were published between 2014 and 2021. Articles and abstracts included in our analysis originated from 5 different countries including Egypt, India, Indonesia, Iran and Vietnam that are all in the LMIC WHO classification.^16^, ^17^, ^18^, ^19^, ^20^, ^21^, ^22^, ^23^, ^24^, ^25^, ^26^, ^27^, ^28^, ^29^, ^30^ No publications from LIC were identified. Thirteen studies were retrospective^16^, ^17^, ^18^, ^19^, ^20^, ^21^, ^22^, ^23^^,^^25^, ^26^, ^27^^,^^29^^,^^30^ and 2 were prospective.^24^^,^^28^ Eleven studies had high quality data and 4 had moderate quality (Supplementary Table 1). Additional details on the background of these publications, including the specific number of patients in each study and study period, can be found in Table 2 and Supplementary Table 1.

**Table 2:** Summary of included study demographics for mechanical thrombectomy outcomes in lower-middle income countries.

Authors	Year	Country	WHO Region	Income Level*	Study Period	Study Design	Center Inclusion	Total Number of Patients
Articles								
Huded et al^16^	2014	India	South-East Asia	Lower-Middle	2010–2012	retrospective	single center	30
Mansour et al^18^	2017	Egypt	Eastern Mediterranean	Lower-Middle	2011–2016	retrospective	single center	113
Luu et al^17^	2020	Vietnam	Western Pacific	Lower-Middle	2017–2018	retrospective	single center	73
Phuoc et al^21^	2020	Vietnam	Western Pacific	Lower-Middle	2017	retrospective	single center	37
Mesiano et al^19^	2021	Indonesia	South-East Asia	Lower-Middle	2017–2020	retrospective	single center	15, MT 14, IVT/MT
Ngoc et al^20^	2021	Vietnam	Western Pacific	Lower-Middle	2009–2017	retrospective	multicenter	269
Vibha et al^22^	2022	India	South-East Asia	Lower-Middle	2017–2019	retrospective	single center	221
***Abstracts***								
Nagesh et al^25^	2016	India	South-East Asia	Lower-Middle	2012–2016	retrospective	single center	28
Salvadeeswaran et al^27^	2016	India	South-East Asia	Lower-Middle	2015	retrospective	single center	13
Fadli^24^	2017	Indonesia	South-East Asia	Lower-Middle	–	prospective	single center	6
Pishjoo et al^26^	2019	Iran	Eastern Mediterranean	Lower-Middle	2018–2019	retrospective	single center	114
Subir et al^28^	2019	India	South-East Asia	Lower-Middle	–	prospective	single center	10
Banga et al^23^	2020	India	South-East Asia	Lower-Middle	2018–2019	retrospective	single center	13
Viet Phuong et al^30^	2020	Vietnam	Western Pacific	Lower-Middle	2018–2019	retrospective	single center	36
Tran et al^29^	2021	Vietnam	Western Pacific	Lower-Middle	2019–2020	retrospective	single center	120

Abbreviations: WHO, World Health Organization; mg/kg, milligram per kilogram; MT, mechanical thrombectomy; IVT, intravenous thrombolysis (tissue plasminogen activator); -, data not available.

*Income classification per 2021 World Bank Country Income Classification (https://datahelpdesk.worldbank.org/knowledgebase/articles/906519-world-bank-country-and-lending-groups).

### 3.3 Thrombectomy outcomes

Ten studies provided data on the median or mean age of patients undergoing MT in the LMIC, and it ranged from 49 to 62 years.^16^, ^17^, ^18^, ^19^, ^20^, ^21^^,^^24^^,^^26^^,^^29^ A female-to-male ratio was provided in 6 of 15 studies, and the percentage of women ranged from 13 to 70%.^16^, ^17^, ^18^, ^19^^,^^21^^,^^26^ Nine of 15 studies provided data on the percentage of patients receiving IV tPA, and the rates ranged from 0 to 100%.^16^, ^17^, ^18^, ^19^^,^^21^^,^^24^^,^^28^^,^^30^ In 8 of 15 studies, the distribution of large vessel occlusions for the patient population was described.^16^, ^17^, ^18^, ^19^^,^^21^^,^^24^^,^^26^^,^^27^ Data on comorbidities included Type II diabetes mellitus, hypertension, hyperlipidemia, atrial fibrillation, and smoker status, and specific numbers are shown in Table 3. Median and mean NIHSS presentation scores for patients were provided in 10 of 15 studies and ranged from 13 to 20.^16^, ^17^, ^18^, ^19^, ^20^, ^21^^,^^26^^,^^28^, ^29^, ^30^ Symptom onset-to-needle puncture time for MT was provided in 5 studies and ranged from 200 to 300 min.^18^^,^^19^^,^^24^^,^^26^ Symptom onset to recanalization time was provided in 3 studies and ranged from 251 to 454 min.^17^^,^^19^ Additional specifics to the aforementioned described data can be found in Table 3.

**Table 3:** Summary of patient demographics in included thrombectomy studies from lower-middle income countries.

Study	Age mean ± SD or median	Female N (%)	IV-tPA given N (%)	Occlusion location N (%)	Type II DM N (%)	HTN N (%)	HLD N (%)	A-Fib N (%)	Smoker N (%)	NIHSS at presentation mean or median^a^	Onset to Needle Time (mins) mean or median^a^	Onset to Recanalization Time (min) mean or median^a^
Full Texts												
Huded et al, 2014^16^	mean, 49.53	–	0 (0.0)	MCA - 16 (53.0) ICA - 10 (33.3) BA - 4 (13.3)	–	–	–	–	–	mean, 20.4	–	–
Mansour et al, 2017^18^	62 ± 11.73	58 (51.3)	8 (7.0)	MCA M1 - 33 (29.2) MCA M2 - 12 (10.6) Carotid T - 17 (15) Total Carotid - 27 (23.9) BA - 21 (18.6) PCA - 2 (1.7) ACA - 1 (0.9)	85 (75.2)	61 (53.9)	85 (75.2)	52 (46.0)	–	16.7 ± 3.2	208.55 ± 53.49	–
Luu et al, 2020^17^	61.29 ± 14.49	35 (47.9)	21 (28.8)	MCA - 28 (38.4) ICA - 33 (45.2) BA - 10 (13.7) PCA - 2 (2.7)	–	24 (32.9)	–	36 (49.3)	–	17.1 ± 5.3	–	251.68 ± 104.70
Phuoc et al, 2020^21^	61.4 ± 13.4	26 (70.3)	13 (35.1)	MCA - 19 (51.4) ICA- 13 (35.1) BA - 5 (13.5)	–	–	–	–	–	17.3 ± 6.9	–	–
Mesiano et al, 2021^19^ (MT group)	median, 55 (38–79)	8 (57.1)	0 (0.0)	MCA M1 - 5 (33.3) MCA M2 - 3 (20.0) ICA - 4 (26.7) BA - 3 (20.0)	5 (33.0)	8 (53.3)	4 (26.7)	5 (33.3)	8 (53.3)	median, 13	median, 360	median, 454
Mesiano et al, 2021^19^ (IVT/MT group)	median, 56.50 (37–85)	2 (13.3)	14 (100.0)	MCA M1 - 5 (35.7) MCA M2 - 7 (50.0) ICA - 2 (14.3)	3 (21.4)	9 (64.3)	1 (7.1)	2 (14.3)	6 (42.9)	median, 13	median, 358	median, 414
Vibha et al, 2022^22^	–	–	–	–	–	–	–	–	–	–	–	–
Ngoc et al, 2021^20^	62.14 ± 13.3	–	–	–	–	–	–	–	–	16.2 ± 7.85	–	–
***Abstracts***												
Nagesh et al, 2016^25^	–	–	–	–	–	–	–	–	–	–	–	–
Salvadeeswaran et al, 2016^27^	–	–	–	MCA M1 – 8 (61.5) MCA M2 – 2 (15.4) ICA – 2 (15.4) Posterior – 1 (7.7)	–	–	–	–	–	–	–	–
Fadli, 2017^24^	mean, 66.6	–	4 (66.7)	MCA M1 – 3 (50.0) Carotid T – 3 (50.0)	–	–	–	–	–	–	mean, 348.6	–
Pishjoo et al, 2019^26^	55.9 ± 13.5	50 (44.0)	–	MCA – 57 (50.0) ICA – 49 (43.0) BA – 8 (7.0)	–	–	–	–	–	16.5 ± 5.1	336 ± 138	–
Subir et al, 2019^28^	–	–	2 (20.0)	–	–	–	–	–	–	mean, 16	–	–
Banga et al, 2020^23^	–	–	–	–	–	–	–	–	–	–	–	–
Viet Phuong et al, 2020^29^			4 (11.1)	–	–	–	–	–	–	median, 13	–	–
Tran et al, 2021^29^	64.4 ± 11.4	–	–	–	–	–	–	–	–	median, 15	–	–

Abbreviations and Acronyms: min, minutes; IV-tPA, intravenous tissue plasminogen activator; HTN, hypertension; HLD, hyperlipidemia; A-Fib, atrial fibrillation; NIHSS, National Institute of Health Stroke Scale; MCA, middle cerebral artery; -, data not available; PCA, posterior cerebral artery; ACA, anterior cerebral artery; ICA, internal carotid artery; BA, basilar artery; MT; mechanical thrombectomy; IVT, intravenous thrombolysis.

a Standard deviation and interquartile range included if specified in the publication.

### 3.4 Meta-analysis of thrombectomy outcomes

**Fig. 2:**
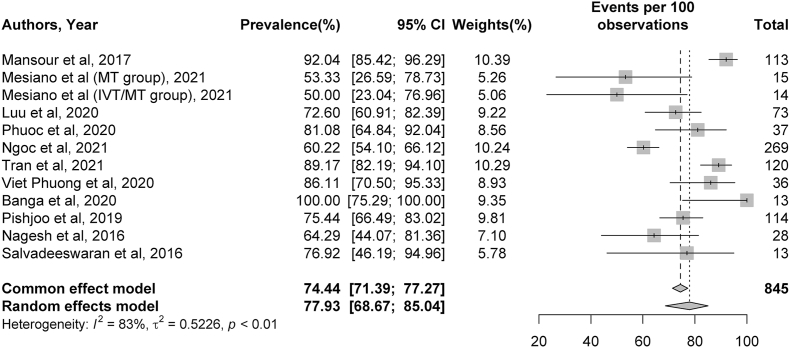
Meta-analysis of Thrombolysis in Cerebral Infarction (TICI) grade 2b or 3 recanalization rates. Plots weighted for sample size show the rates of TICI 2b or 3 recanalization after thrombectomy. The article written by Mesiano et al (Cerebrovasc Dis Extra 2021; 11(2)72–76; reference 18) was separated into 2 studies because it described two distinct thrombectomy populations (one receiving intravenous thrombolysis [IVT] and MT and another receiving MT alone). CI, confidence interval.

**Fig. 3:**
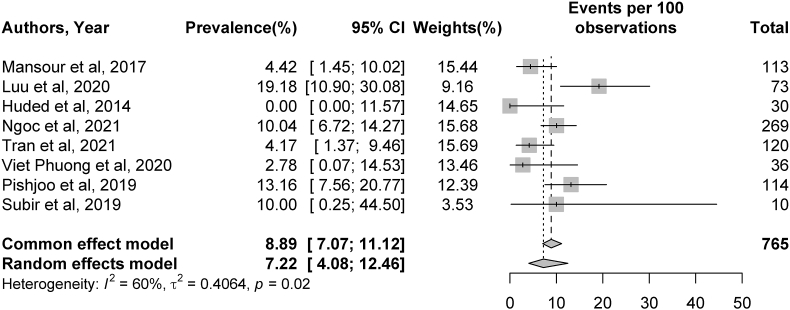
Meta-analysis of complication rates of symptomatic intracranial hemorrhage. Plots weighted for sample size show rates of symptomatic intracranial hemorrhage as a complication post-stroke ranging from 2 to 19%. CI, confidence interval.

**Fig. 4:**
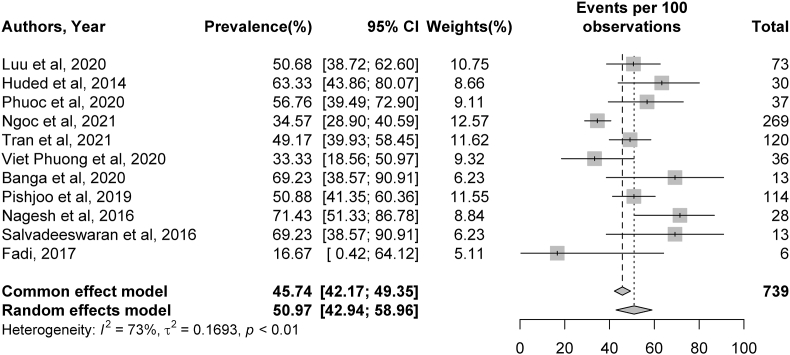
Meta-analysis of rates of modified Rankin Scale (mRS) 0 to 2 outcomes at 90 days. Plots weighted for sample size show rates of mRS 0 to 2 outcomes at 90 days post-stroke ranging from 16 to 71%. CI, confidence interval.

**Fig. 5:**
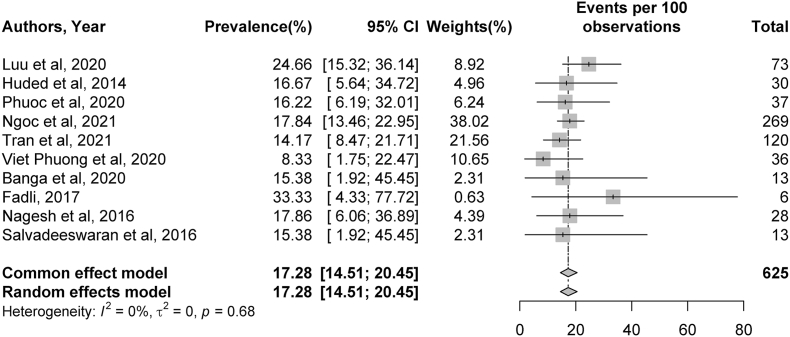
Meta-analysis of 90-day mortality rates post-stroke. Plots weighted for sample size show the mortality rates at 90 days post-stroke ranging from 8 to 24%. CI, confidence interval.

Thirteen studies presented data on recanalization after MT, and the pooled rate of TICI 2b/3 recanalization was 77.93%.^17^, ^18^, ^19^, ^20^, ^21^^,^^23^^,^^25^, ^26^, ^27^^,^^29^^,^^30^ Symptomatic ICH complications were present in 8 studies,^16^, ^17^, ^18^^,^^20^^,^^26^^,^^28^, ^29^, ^30^ and the rate of periprocedural sICH following MT was 7.22%. Based on 11 studies,^16^^,^^17^^,^^20^^,^^21^^,^^23^, ^24^, ^25^, ^26^, ^27^^,^^29^^,^^30^ 50.97% of patients had an mRS score of 0–2 at 90 days post-stroke. The mortality rate, based on 10 studies, was 17.28% at 90 days post-stroke.^16^^,^^17^^,^^20^^,^^21^^,^^23^, ^24^, ^25^^,^^27^^,^^29^^,^^30^ Additional details, including specific CIs of the percentages described above, can be found in Fig. 2, Fig. 3, Fig. 4, Fig. 5.

Meta-analysis was conducted for rates of TICI2b/3 recanalization, sICH complications, 90-day mRS outcomes, and 90-day mortality rates. Significant heterogeneity was identified across studies for rates of TICI 2b/3 recanalization (I^2^ = 83%, *p* < 0.01), sICH (I^2^ = 60%, *p* = 0.02), and 90-day mRS outcomes (I^2^ = 73%, *p* < 0.01). No significant heterogeneity was present among studies describing 90-day mortality rates (I^2^ = 0%, *p* = 0.68). Additional details can be found in Fig. 2, Fig. 3, Fig. 4, Fig. 5.

## 4 Discussion

Clot retrieval and thrombectomy first entered the endovascular field in 2004 with the Merci retrieval system (Inova Neurosciences, Fairfax, VA 22031), and a clear benefit over medical thrombolysis was not evident.^31^ This was followed by several randomized control trials that failed to clarify the equipoise of thrombectomy versus thrombolysis.^32^^,^^33^ With the advancement of endovascular technique and clot-retrieval devices including stent retrievers, this equipoise disappeared. Key randomized trials were completed with stent retrievers and were associated with improved success and better patient outcomes than medical management and thrombolysis.^3^, ^4^, ^5^, ^6^, ^7^ However, all the trials were conducted in higher level income countries including the Netherlands,^3^ United States,^5^^,^^7^ Australia,^4^ New Zealand,^4^ Spain,^6^ Canada,^5^, ^6^, ^7^ Germany,^6^ and Ireland.^5^ Although the global mortality rate for stroke decreased with advancing treatment strategies and better patient care, the decline in the mortality rate has been higher in LIC and LMIC compared to middle and high income countries.^34^ As mentioned, up to 87% of mortality and 81% of disability related to stroke are from LIC and LMIC.^8^ It is important to consider how the outcomes of these trials are so pivotal but may not be as applicable in LIC and LMIC. This disparity may be due to several reasons, including the lack of established stroke practice and care guidelines.^35^ A worldwide survey highlighted a “460-fold disparity between highest and lowest nonzero” MT-capable regions in which low-income countries had “88% lower MT access compared to high-income countries.^9^ More specifically, Baatiema et al conducted a review of stroke management in Africa and concluded there was “low applicability and uptake of evidence-based stroke care in lower middle income countries” based on studies that described only medical thrombolytic therapy.^36^ Another reason may be the lack of an academic and research structure to improve patient care, as Pandian et al showed in their review of stroke care services in LIC and middle income countries. Those authors found few articles that described feasible stroke care service models and primarily highlighted a clear lack in outcomes data and recommended prioritization of additional research for implementation of locally applicable stroke care services.^11^ This difference in research and pathology bibliometrics among varying income level countries is seen not only in the stroke literature but also in the oncology literature.^37^

The aforementioned points question the applicability of thrombectomy and its alleged improvement in stroke outcomes in LIC and LMIC; however, to our knowledge, no review of the literature has been conducted looking at thrombectomy outcomes in these countries. In our literature search, we identified 15 total studies describing thrombectomy outcomes in LMIC countries. The rate of TICI 2b/3 recanalization (77.93%) was similar to the rates seen in the SOLITAIRE™ FR With the Intention For Thrombectomy (SWIFT)^38^ and Trevo versus Merci retrievers for thrombectomy revascularization of large vessel occlusions in acute ischaemic stroke (TREVO 2)^39^ trials, as well an article detailing the aspiration thrombectomy technique.^40^ The rates of sICH in our analysis were slightly higher than those in the analysis conducted by the Highly Effective Reperfusion Evaluated in Multiple Endovascular Stroke (HERMES) collaborators (7.22% vs. 4.4%, respectively).^41^ Mortality at 90 days was also higher at 17.28% compared to 15.3% seen in the intervention cohort of the HERMES analysis.^41^ The percentage of patients with an mRS 0–2 score after thrombectomy was higher at 50.97% in patients from LIC and LMIC articles compared to the HERMES analysis (46.0%). Overall, these three metrics were within 5% of those seen in the HERMES analysis. The professions performing thrombectomy aren't explicitly stated in the literature, but it is likely neurosurgeons, vascular neurologists, or interventional neuroradiologists trained in endovascular procedures. These specialized trainings are mainly accessible in certain programs in wealthier nations. Foreign neurosurgeons, whether trained internationally or in the US, more commonly pursue endovascular or skull base fellowships within the US.^42^ This could offer similar endovascular expertise to neurosurgeons across different income level countries, potentially explaining the similar thrombectomy outcomes in this study. In lower income countries, endovascular facilities might only be accessible at tertiary or private hospital setting where patient care is more comprehensive. These centers, performing numerous thrombectomies, may generate higher and better quality publications meeting journal standards. This aspect further clarifies the findings on thrombectomy in this study.

Although the outcomes were overall similar between the major trials and published thrombectomy outcomes from the LMIC countries, it is important to highlight not only the small number of articles identified in our screening but also the few countries of origin of these studies. Among the 31 LIC and 51 LMIC countries, after screening more than 8000 search hits, only 15 studies from 5 LMIC countries were identified, including Egypt, India, Indonesia, Iran, and Vietnam. This prompts us to ask why there are no published results from the other 77 countries. Other questions include: Is there a lack of adaptation in thrombectomy techniques in these countries? Is there a lack of trained personnel to educate, teach, and execute thrombectomy there? Are sufficient financial resources available to afford the newer generation of stent retrievers and aspiration catheters? As previously mentioned, is this scarce finding of literature highlighting a lack of academic and research infrastructure? A multitude of factors may account for the lack of more publications reporting thrombectomy outcomes. Sundar et al looked into the disparity of stroke care and concluded that the access and time to MT was a critical factor in worse stroke outcomes in India.^43^

An important initiative, Mission Thrombectomy 2020, was started by the Society of Vascular and Interventional Neurology with the aim of increasing access to stroke thrombectomy globally.^43^ We hope that this manuscript highlights the results of the current literature on thrombectomy in LIC and LMIC and points out that future efforts are needed to improve global access and reporting of stroke care including thrombectomy in LIC and LMIC countries.

This study has several limitations. There is significant heterogeneity among patient populations in the publications identified. The goal of this study was to present available thrombectomy outcomes in LIC and LMIC. To accomplish this, we maintained broad inclusion criteria and did not exclude publications based on their methodological quality, thus including both abstracts and full text articles. Therefore, strict inclusion criteria based on a standardized study methodology, uniformity of thrombectomy technique, individual inclusion criteria were not possible. Furthermore, patient level data were not available, and detailed patient data presentations were not possible. Not all publications presented the data points of interest, including complication rates, 90-day mortality rates, and 90-day mRS outcomes. We must also consider that there was heterogeneity in the way in which the data from all publications were presented. Although these limitations exist, we hope they also highlight the lack of literature and description of thrombectomy in LIC and LMIC.

## 5 Conclusion

Only 5 LMIC countries have published data on thrombectomy for ischemic stroke, highlighting a significant void in the literature and potentially a lack of accessibility to stroke thrombectomy care in LIC and LMIC. Global prioritization from the neuroendovascular and neurological community is needed to help improve this void.

## Funding

This research did not receive any specific grant from funding agencies in the public, commercial, or not-for-profit sectors.

## CRediT authorship contribution statement

**Jaims Lim:** Writing – review & editing, Writing – original draft, Visualization, Validation, Supervision, Project administration, Methodology, Investigation, Conceptualization. **Alexander O. Aguirre:** Writing – review & editing, Writing – original draft, Visualization, Validation, Methodology, Investigation, Formal analysis, Data curation, Conceptualization. **Abbas Rattani:** Writing – review & editing, Writing – original draft, Visualization, Validation, Methodology, Investigation, Conceptualization. **Ammad A. Baig:** Writing – review & editing, Writing – original draft, Visualization, Validation, Supervision, Project administration, Methodology, Investigation, Conceptualization. **Andre Monteiro:** Writing – review & editing, Writing – original draft, Visualization, Validation, Supervision, Project administration, Methodology, Investigation. **Cathleen C. Kuo:** Writing – original draft, Visualization, Validation, Investigation, Formal analysis, Data curation. **Manhal Siddiqi:** Writing – original draft, Visualization, Validation, Investigation, Data curation. **Justin Im:** Writing – original draft, Visualization, Validation, Investigation, Data curation. **Steven B. Housley:** Writing – review & editing, Writing – original draft, Visualization, Validation, Supervision, Project administration, Methodology, Investigation. **Matthew J. McPheeters:** Writing – review & editing, Writing – original draft, Visualization, Validation, Methodology, Investigation. **Shiau-Sing K. Ciecierska:** Writing – review & editing, Writing – original draft, Visualization, Validation, Investigation, Formal analysis, Data curation. **Vinay Jaikumar:** Writing – review & editing, Writing – original draft, Visualization, Validation, Methodology, Investigation, Formal analysis, Data curation. **Kunal Vakharia:** Writing – review & editing, Writing – original draft, Visualization, Validation, Supervision, Methodology, Investigation. **Jason M. Davies:** Writing – review & editing, Writing – original draft, Visualization, Validation, Supervision, Methodology, Investigation. **Kenneth V. Snyder:** Writing – review & editing, Writing – original draft, Visualization, Validation, Supervision, Methodology, Investigation. **Elad I. Levy:** Writing – review & editing, Writing – original draft, Visualization, Validation, Supervision, Methodology, Investigation. **Adnan H. Siddiqui:** Writing – review & editing, Visualization, Validation, Supervision, Resources, Project administration.

## Declaration of competing interest

The authors declare that they have no known competing financial interests or personal relationships that could have appeared to influence the work reported in this paper.
