# Supplementary material for: Thrombectomy outcomes for acute ischemic stroke in lower-middle income countries: A systematic review and analysis
Source: World Neurosurg X. 2024 Mar 5;23:100317. doi: 10.1016/j.wnsx.2024.100317 (PMC10950731; doi:10.1016/j.wnsx.2024.100317)
Supplement: Multimedia component 1 [file mmc1.docx]

**Supplementary Table 1: Quality Assessment**

| **Study** | **Prospective design** | **Stroke severity** | **Procedural (Thrombectomy ±thrombolysis) details** | **Peri-and post- procedural complications** | **Angiographic outcomes** | **Clinical outcomes** | **Long term follow up**  **(≥90 days)** | **Total** | **Quality** |
| --- | --- | --- | --- | --- | --- | --- | --- | --- | --- |
| Huded 2014 | No | Yes | Yes | Yes | Yes | Yes | Yes | 6 | High |
| Luu 2020 | No | Yes | Yes | Yes | Yes | Yes | Yes | 6 | High |
| Mansour 2017 | No | Yes | Yes | Yes | Yes | Yes | Yes | 6 | High |
| Mesiano 2021 | No | Yes | Yes | Yes | Yes | Yes | No | 5 | High |
| Ngoc 2021 | No | Yes | Yes | Yes | Yes | Yes | Yes | 6 | High |
| Phuoc 2020 | No | Yes | Yes | Yes | Yes | Yes | Yes | 6 | High |
| Vibha 2022 | No | Yes | Yes | Yes | Yes | Yes | No | 5 | High |
| Banga 2020 | No | No | Yes | No | Yes | Yes | Yes | 4 | Moderate |
| Fadli 2017 | Yes | Yes | Yes | No | No | Yes | Yes | 5 | High |
| Nagesh 2016 | No | No | Yes | Yes | Yes | Yes | No | 4 | Moderate |
| Pishjoo 2019 | No | Yes | Yes | Yes | Yes | Yes | Yes | 6 | High |
| Salvadeeswaran 2016 | No | No | No | No | Yes | Yes | Yes | 3 | Moderate |
| Subir 2019 | Yes | No | Yes | Yes | No | Yes | No | 4 | Moderate |
| Tran 2021 | No | No | Yes | Yes | Yes | Yes | Yes | 5 | High |
| Phuong 2020 | No | Yes | Yes | Yes | Yes | Yes | Yes | 6 | High |

**Scoring**: Score ≤2, Low quality; Score 3-4, Moderate quality; Score ≥5, High quality
